# Enhanced Conversion Efficiency of III–V Triple-junction Solar Cells with Graphene Quantum Dots

**DOI:** 10.1038/srep39163

**Published:** 2016-12-16

**Authors:** Tzu-Neng Lin, Svette Reina Merden S. Santiago, Jie-An Zheng, Yu-Chiang Chao, Chi-Tsu Yuan, Ji-Lin Shen, Chih-Hung Wu, Cheng- An J. Lin, Wei-Ren Liu, Ming-Chiang Cheng, Wu-Ching Chou

**Affiliations:** 1 Department of Physics and Center for Nanotechnology, Chung Yuan Christian University, Chung-Li 32023, Taiwan; 2 Institute of Nuclear Energy Research, P. O. Box 3-11, Lung-Tan 32500, Taiwan; 3 Department of Biomedical Engineering, Chung Yuan Christian University, Chung-Li 32023, Taiwan; 4 Department of Chemical Engineering, Chung Yuan Christian University, Chungli, 32023, Taiwan; 5 Department of Electrophysics, National Chiao Tung University, Hsinchu 300, Taiwan

## Abstract

Graphene has been used to synthesize graphene quantum dots (GQDs) via pulsed laser ablation. By depositing the synthesized GQDs on the surface of InGaP/InGaAs/Ge triple-junction solar cells, the short-circuit current, fill factor, and conversion efficiency were enhanced remarkably. As the GQD concentration is increased, the conversion efficiency in the solar cell increases accordingly. A conversion efficiency of 33.2% for InGaP/InGaAs/Ge triple-junction solar cells has been achieved at the GQD concentration of 1.2 mg/ml, corresponding to a 35% enhancement compared to the cell without GQDs. On the basis of time-resolved photoluminescence, external quantum efficiency, and work-function measurements, we suggest that the efficiency enhancement in the InGaP/InGaAs/Ge triple-junction solar cells is primarily caused by the carrier injection from GQDs to the InGaP top subcell.

III–V compound multi-junction tandem solar cells are promising as the most efficient photovoltaic devices among a diversity of solar cell structures. To date, the best III–V compound triple-junction and four-junction solar cells have attained conversion efficiencies of up to 44.4 and 46% under concentrated light, respectively^1^,^2^. Such high-efficiency multi-junction solar cells can be utilized in space and in terrestrial applications and greatly reduce the cost of solar electricity generation. To further increase the conversion efficiency it is necessary to enhance light absorption, increase carrier collection, or reduce the energy loss due to thermalization. Several strategies have been proposed to increase the conversion efficiency in III–V multi-junction solar cells^3^,^4^,^5^,^6^. For example, a new material engineering technique has been developed for maintaining high thermal homogeneity at the wafer surface, which expands the bandgap engineering possibilities for improving the efficiency in III–V multi-junction solar cells^4^. Also, metal nanoparticles have been introduced on the illuminated surface of III–V multi-junction solar cells for producing strong light scattering and light trapping, which is beneficial for carrier generation in the absorbing region^5^,^6^. A 15% increase of conversion efficiency has been implemented by introducing Au nanoclusters on top of the III–V multi-junction solar cells^5^.

Graphene quantum dots (GQDs), composed of few layers of graphene with the nano-sized lateral dimensions, are a new class of graphene nanostructures. GQDs have been extensively exploited due to their peculiar properties such as low toxicity, strong fluorescence, high surface area, large solubility, and tunable band gaps^7^,^8^,^9^,^10^. Recently, GQDs have shown great promise in organic or organic-inorganic hybrid photovoltaic applications^7^,^8^,^9^. GQDs have been used as electron acceptors in a P3HT-based solar cell, wherein GQDs improve the electron transport in the active layer and the device achieve a 1.28% conversion efficiency^9^. GQDs have also been used as an intermediate buffer layer to form a cascade alignment of energy levels in organic-inorganic hybrid solar cells, facilitating the charge carrier transport and increasing the cell efficiency^7^,^8^. In addition, a combination of GQDs and ZnO nanowires have been demonstrated as a solar harvesting material in solid-state solar cells^10^. The photovoltaic performance made up by ZnO/GQD-based cells showed a value of high open circuit voltage (V_OC_) of ~0.8 V and an internal quantum efficiency of 87%. Very recently, GQDs have been deposited as down-converters on silicon-related solar cells, improving the short circuit current (I_sc_) and the conversion efficiency by 2.94–6.1% and 2.7–14.9%, respectively^11^,^12^.

It is well known that GQDs exhibit a lower work function (~3.7 eV) than that of many semiconductors such as GaAs, GaN, Si, and TiO_2_^13^,^14^. This property enables the charge carriers in GQDs to transfer into the semiconductors since the LUMO level of GQDs is higher than that of the conduction band of the semiconductors. Upon photoexcitation, GQDs can donate the carriers into the semiconductors through the GQD/semiconductor interface, influencing the optical and electrical properties of the semiconductors. Recently, GQDs have been utilized as photoinduced dopants for donating photogenerated electrons to semiconductors and enhance the semiconductor emission and photocurrent^15^,^16^. In a semiconductor solar cell, an increase of carriers in the active layer corresponds to an enhancement of the carrier lifetime and diffusion length, which is beneficial for the collection efficiency. In other words, by depositing GQDs on the surface of semiconductor solar cells, of which the work function of the semiconductor surface is larger than that of GQDs, the carriers in semiconductor solar cells can increase owing to the carrier injection. Thus, GQDs can donate carriers into the active layer of semiconductor solar cells and the efficiency of solar cells can enhance accordingly. Based on this argument, GQDs could effectively improve the photovoltaic characteristics of the III–V solar cells since most III–V semiconductor materials have a larger work function than that in GQDs. In this paper, we synthesized GQDs from graphene flakes in ethanol by pulse laser ablation. This synthesis produces GQDs with an average size of 3.8 nm from analysis of the TEM image. By depositing the as-synthesized GQDs on the InGaP/InGaAs/Ge triple-junction solar cell, GQDs significantly enhance the short-circuit current, the fill factor, and the conversion efficiency in the InGaP/InGaAs/Ge triple-junction solar cell. On the basis of the photoluminescence (PL), time-resolved PL, external quantum efficiency (EQE), and work function measurements, the mechanism that causes enhancement of conversion efficiency is discussed.

## Materials and Methods

### Fabrication of Triple Junction Solar Cells

**Figure 1:**
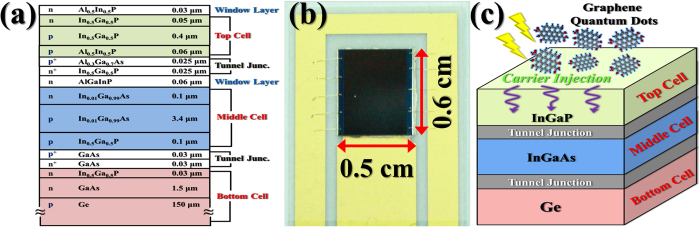
(**a**) Schematic illustration of the investigated InGaP/InGaAs/Ge triple-junction solar cell. (**b**) Photograph of the InGaP/InGaAs/Ge triple-junction solar cell. (**c**) Schematic representation of the carrier injection from graphene quantum dots (GQDs) to the InGaP/InGaAs/Ge triple-junction solar cell.

The III–V triple-junction solar cells investigated were composed of monolithic cascade-type InGaP/InGaAs/Ge triple-junctions connected in series without anti-reflection coatings. The details of the sample structure are described elsewhere^5^. Briefly, the top In_0.51_Ga_0.49_P, middle In_0.01_Ga_0.99_As, and bottom Ge subcells were all lattice-matched and grown on a p-type Ge substrate by metal-organic chemical vapor deposition (MOCVD). The InGaP subcell was connected to the InGaAs subcell by a p-AlGaAs/n-InGaP tunnel junction. The InGaAs subcell was then connected to the Ge subcell by a p-GaAs/n-GaAs tunnel junction. The size of InGaP/InGaAs/Ge triple-junction solar cells is 0.5 cm × 0.6 cm in rectangular shape with the active area of ~0.3 cm^2^). The schematic and photograph of the typical InGaP/InGaAs/Ge triple-junction solar cell investigated are shown in Fig. 1a and b, respectively.

### Synthesis of GQDs

The GQDs investigated were synthesized in ethanol solution by the pulsed laser ablation^16^. The raw graphene powder for synthesis of GQDs was purchased from the *Graphene Supermarket (USA*). A 0.03 g of graphene powders was added to ethanol (7 ml) and incorporated using a vortex mixer thoroughly. The solution (600 μl) was placed in a quartz cell and then allocated on a rotating stage with an angular velocity of 80 rpm. An optical parametric oscillator (OPO) laser (415 nm, 10 Hz, 10 ns) was used to ablate the sample for 5 min. The power of the OPO laser was controlled under the fluence of 2.58 J/cm^2^. After being treated to laser ablation, the resulting suspension was centrifuged and filtered through a 0.22 μm syringe filters. To examine the morphologies of GQDs, a transmission electron microscopy (TEM) (JEOL JEM-2100F) was carried out.

### Characterization

To investigate the effect of GQDs on the performance of the triple-junction solar cell, the GQDs were deposited on the cell surface by drop casting (Fig. 1b). Each 5 μl droplet of the GQD solution was pipetted onto the surface of the top subcell with an area of ~0.3 cm^2^. For the current-voltage (I–V) characteristics, four cell samples have been measured under the same conditions. The I–V characteristics of four individual cells were measured using a Keithley 2400 source meter and the photovoltaic I-V characteristics were made under AM1.5 global solar spectrum with one-sun total intensity (100 mW cm^−2^). The external quantum efficiency (EQE) spectra of the InGaP/InGaAs/Ge triple-junction solar cells were measured using a spectral response measurement system (Enlitech, QE-R). To probe each subcell’s EQE, a combination of red/infrared, blue/infrared, and green/red LEDs have been used as light biases. The time-resolved PL was performed with a solid-state pulsed laser (347 nm, 70 MHz, 250 fs) using the technique of time-correlated single-photon counting (TCSPC). The collected luminescence was dispersed by a 0.75 m spectrometer and detected with a high-speed photomultiplier tube (PMT). The instrument response of the time-correlated single photon counting system is around 250 ps. The work function measurements were carried out using a Kelvin probe system (KP Technology, KP020).

## Results and Discussion

**Figure 2:**
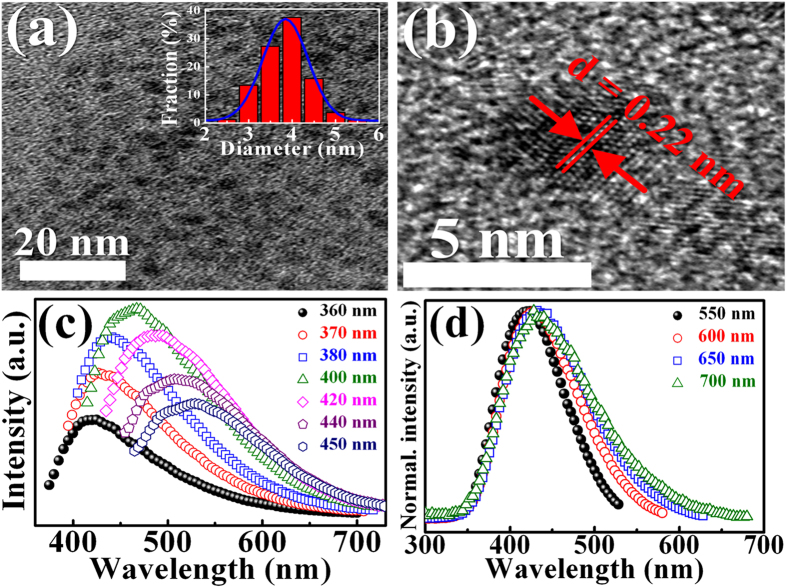
(**a**) TEM image of GQDs. The inset shows the size distribution of the GQDs. (**b**) HRTEM image of an individual GQD. (**c**) Excitation-dependent PL spectra of the GQDs. (**d**) Emission-dependent PLE spectra of the GQDs.

The GQDs synthesized were subjected to different characterizations which are seen in Fig. 2a–d. The TEM image of the typical GQDs is shown in Fig. 2a, indicating a monodispersed distribution. The inset shows the size distribution of the particles, revealing an average size and a full width at half maximum (FWHM) of ~3.8 nm and ~1.4 nm, respectively. Figure 2b shows the high-resolution TEM (HRTEM) image of a single GQD. The microstructure in HRTEM reveals a crystalline structure with an interplanar spacing of 0.22 nm, corresponding to the (102) lattice fringe of graphene^17^. Figure 2c shows PL spectra of the synthesized GQDs obtained with different excitation wavelength. By excitation with wavelengths of 360 to 450 nm, the PL peak is shifted from 420 to 530 nm. A similar dependence of PL emission on excitation wavelength has been observed in previous studies involving GQDs^18^,^19^. The shift in the PL peak wavelength has been attributed to different emitting species or the localization of electron-hole pairs due to the isolated sp^2^ clusters within the sp^3^ matrix^20^. Figure 2d shows PL excitation (PLE) spectra for different emission peaks in the synthesized GQDs, indicating a main peak located at around 420–450 nm. The excitation-dependent characteristics of PL and PLE in GQDs are beneficial for some applications in optoelectronic devices.

**Figure 3:**
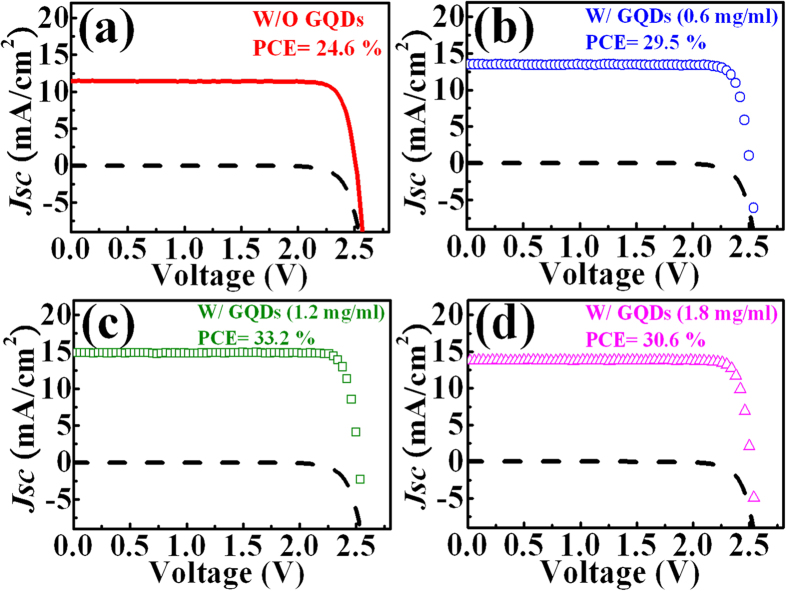
The photovoltaic J–V characteristics of triple-junction solar cells with various concentration of GQDs. (**a**) 0 (solid line), (**b**) 0.6 (open circles), (**c**) 1.2 (open squares), and (**d**) 1.8 mg/ml (open triangles). The dashed lines represent the J-V characteristics of the cell under dark condition. The power conversion efficiency (PCE) of the cells with various concentration of GQDs are also shown.

**Figure 4:**
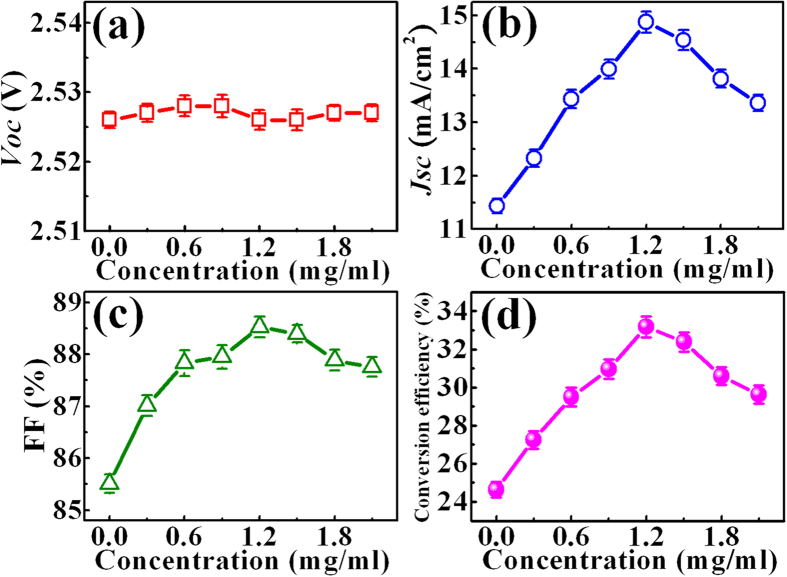
(**a**) Open-circuit voltage, (**b**) short-circuit current density, (**c**) fill factor, and (**d**) conversion efficiency of the GQD/cell composite as a function of the GQD concentration.

Figure 3a shows the I–V characteristics of the InGaP/InGaAs/Ge triple-junction solar cells under dark and illuminated conditions. The I–V curve with the solar irradiance shows a clear down shift to that without illumination, indicating J_sc_ = 11.4 mA/cm^2^, V_oc_ = 2.53 V, FF = 85.5, and η = 24.6%, where J_sc_, V_oc_, FF, and η are short-circuit current density, open-circuit voltage, the fill factor, and conversion efficiency, respectively. Figure 3b–d shows the I–V characteristics of the InGaP/InGaAs/Ge triple-junction solar cells under dark and illuminated conditions after deposition of different GQD concentrations. Figure 4a–c exhibit V_oc_, J_sc_, and FF as a function of the GQD concentration, respectively. After introduction of GQDs, no significant difference in V_oc_ was observed, while an enhancement of J_sc_ was clearly observed with the increase of the GQD concentration from 0 to 1.2 mg/ml. The enhanced J_sc_ reaches its maximum value of 14.9 mA/cm^2^ at the GQD concentration of 1.2 mg/ml, corresponding to a 31% increase in J_sc_. After the GQD concentration is over 1.2 mg/ml, J_sc_ saturates and decreases. FF of the InGaP/InGaAs/Ge triple-junction solar cell increases also from 85.5 to 88.5 and saturates across the GQD concentration range investigated, as shown in Fig. 4c. η obtained from the above photovoltaic I–V characteristics as a function of the GQD concentration is shown in Fig. 4d. The conversion efficiency of the solar cell without GQDs is 24.4% (obtained from Fig. 4a), which is lower than that of typical III-V triple-junction solar cells^1^,^2^. This is because the investigated cell in our work is the cell without antireflection layer. Similar power conversion efficiencies (e.g. 23.66% and 23.8%) have been reported for the InGaP/InGaAs/Ge triple-junction solar cell without antireflection coating^21^,^22^. A remarkable increase in η is clearly observed as the GQD concentration increases. The η in the InGaP/InGaAs/Ge triple-junction solar cell reaches its maximum value of 33.2% at the GQD concentration of 1.2 mg/ml, corresponding to a 35% enhancement compared to the cell without introduction of GQDs. To our knowledge, the 35% increase in the conversion efficiency is much better than the previous report on the enhancement of InGaP/InGaAs/Ge triple-junction solar cells by introducing other nanoparticles^5^,^21^. Thus, the incorporation of GQDs can be an effective and convenient method for improving the conversion efficiency in InGaP/InGaAs/Ge triple-junction solar cells.

**Figure 5:**
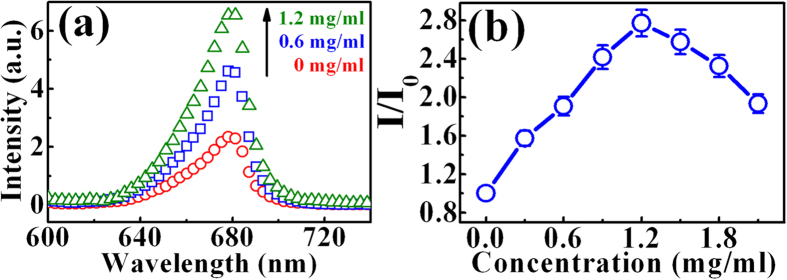
(**a**) PL spectra of the InGaP after incorporation of GQDs with different concentration: 0 (open circles), 0.6 mg/ml (open squares), and 1.2 mg/ml (open triangles). (**b**) The PL intensity ratio of the InGaP with GQDs to that without GQDs as a function of the GQD concentration. The line is a guide to eyes.

To find out the origin of improvement in the solar cell efficiency, the steady-state and time-resolved PL of the InGaP/InGaAs/Ge triple-junction solar cell were investigated. Figure 5a shows the PL spectra of the InGaP/InGaAs/Ge triple-junction solar cells after the incorporation of GQDs with different concentrations. The spectra reveal a PL peak of InGaP located at around 1.9 eV, which is originated from the InGaP top subcell. The observed PL signal is mainly attributed from the p-type base layer in the InGaP top subcell since that layer is the thickest^23^. As shown in Fig. 5a, a clear enhancement of PL intensity in the InGaP top subcell occurs after incorporation of GQDs. Changes of the PL intensity after incorporation of GQDs with different concentrations are shown in Fig. 5b. The PL intensity in the InGaP top subcell increases with increasing the GQD concentration. The maximum PL intensity occurs at the GQD concentration of 1.2 mg/ml, revealing an enhancement factor of ~2.7. The enhancement of PL intensity indicates an increase of the recombined carriers in the InGaP top subcell after introduction of GQDs.

**Figure 6:**
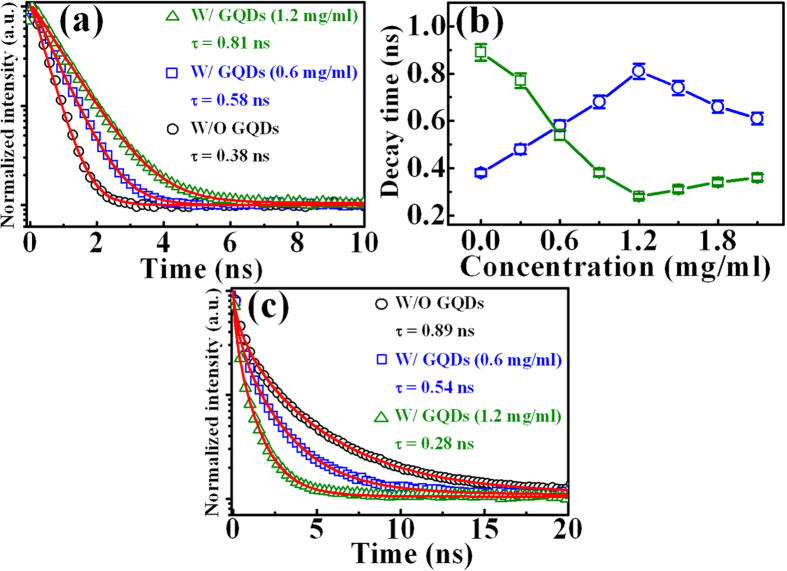
(**a**) The PL decay profiles of the InGaP in the GQD/cell composite with different GQD concentrations: 0(open circles), 0.6 (open squares), and 1.2 mg/ml (open triangles). The solid lines show the fitted curves using equation (1). (**b**) The PL decay times of the InGaP layer (open circles) and GQDs (open squares) in the GQD/cell composite as a function of the GQD concentration. The lines are guides to the eyes. (**c**) The PL decay profiles of the GQDs in the GQD/cell composite with different GQD concentrations: 0 (open circles), 0.6 (open squares), and 1.2 mg/ml (open triangles). The solid lines show the fitted curves using equation (2).

The open squares in Fig. 6a show the PL decay profiles of the InGaP peak in the InGaP top subcell after the incorporation of GQDs with different concentrations. The PL decay transients in the GQD/cell composite decay less as compared to the bare cell. The PL decay curves can be fitted by a single exponential function^24^:


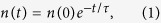


where *n(t*) is the carrier density and *τ* is the PL decay time of the minority carrier. The fitted results are shown in the solid line of Fig. 6a, which are in good agreement with experimental data. The open circles in Fig. 6b show the obtained PL decay times of InGaP in the GQD/cell composite as a function of the GQD concentration. The PL decay time of InGaP increases with the increase of the GQD concentration and reaches its maximum value of 0.82 ns for incorporation of GQDs with concentration of 1.2 mg/ml. After the concentration more than 1.2 mg/ml, the PL decay time begins to decrease. In solar cell physics, the performance of solar cells is strongly dependent on the diffusion length, which is associated with the minority carrier lifetime, i.e., the PL decay time. Thus, the increase of the PL decay time in Fig. 6b confirms the improvement of performance in InGaP/InGaAs/Ge solar cells by the introduction of GQDs.

Figure 6c displays the PL decays of GQDs after incorporation on the InGaP/InGaAs/Ge triple-junction solar cell. The PL decay curves of GQDs in the GQD/cell composite decay more pronouncedly as compared with that in bare GQDs. All the PL decay curves can be fitted by the stretched exponential function^15^:


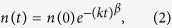


where *k* is the decay rate of carriers and *β* is a dispersive exponent. The fitted results are shown in the solid line of Fig. 6c, in good agreement with the experimental data.

The average decay time in the stretched exponential function is described by ref. 25:


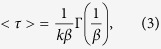


where Γ is the Gamma function. The open circles in Fig. 6b show the obtained PL decay times of GQDs in the GQD/cell composite as a function of the GQD concentration. Obviously, the PL decay time of GQDs decreases with the increase of the GQD concentration. The PL decay time of GQDs reaches its minimum value of 0.28 ns for the introduction of GQDs with the concentration of 1.2 mg/ml. It is noted that the increase of the PL decay times in the InGaP top subcell corresponds well to the decrease of that in GQDs as the GQD concentration increases (Fig. 6b). This implies an occurrence of carrier injection from the GQDs to the InGaP top subcell. The carrier injection may increase the carrier lifetime (diffusion length) which is responsible for the enhanced performance in the InGaP/InGaAs/Ge triple-junction solar cell.

**Figure 7:**
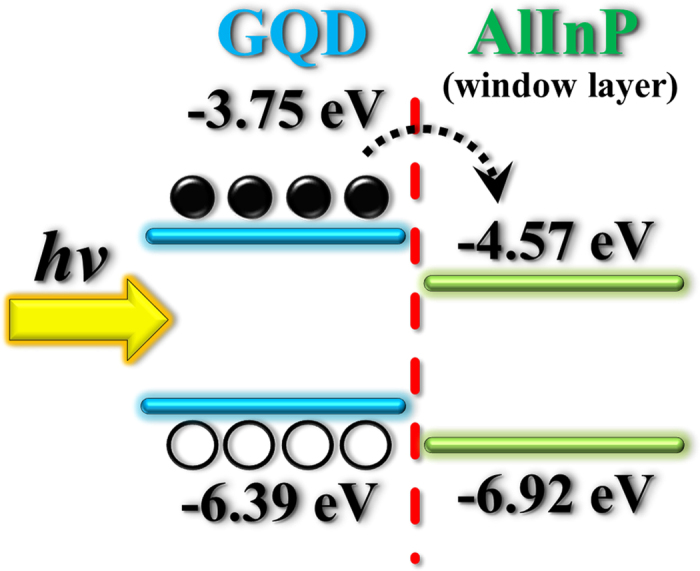
Energy level diagram of AlInP (the window layer of the InGaP top subcell) in contact with GQDs, describing the carrier injection.

To investigate the mechanism of carrier injection, the work function measurements of GQDs and the AlInP layer (the window layer of the InGaP top subcell) were carried out. The contact potential difference (*V*_*CPD*_) between the sample and tip can be associated with the difference in work function between them from the following equation:


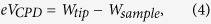


where *W*_*tip*_ and *W*_*sample*_ are work functions of the tip and sample, respectively. By measuring *V*_*CPD*_, the sample work function is obtained as long as the *W*_*tip*_ can be determined. Using Kelvin probe measurements, the work functions of the GQDs and AlInP were estimated to be 3.75 ± 0.02 eV and 4.57 ± 0.03 eV, respectively. According to the work function measurements, the carrier injection from GQDs to InGaP top subcells can be explained as follows. When GQDs are incorporated on the surface of the InGaP top subcell, the photogenerated carriers in GQDs can be easily injected into the AlInP layer because the work function of GQDs (3.75 eV) is smaller than that of the AlInP layer (4.57 eV) (Fig. 7). The carrier injection produces extra carriers into the InGaP top subcell and therefore enhances the carrier density in InGaP/InGaAs/Ge triple-junction solar cells. An enhancement of the carrier density leads to an increase in the carrier lifetime (diffusion length), promoting the collection and conversion efficiencies in solar cells. At higher concentrations of GQDs, more carriers in GQDs would be generated by photoexcitation, producing higher carrier injection rate and more carrier densities in the InGaP/InGaAs/Ge triple-junction solar cell. Thus, the high-concentration GQDs can achieve a higher enhancement in J_SC_, FF and η, as displayed in Fig. 4b–d, respectively.

**Figure 8:**
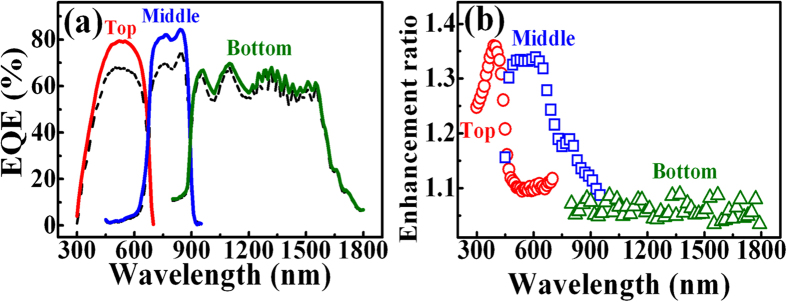
(**a**) External quantum efficiency (EQE) spectra of the InGaP/InGaAs/Ge triple-junction solar cell with (solid line) and without (dashed line) the introduction of GQDs with a concentration of 1.2 mg/ml. (**b**) EQE enhancement ratio of the InGaP/InGaAs/Ge triple-junction solar cell after the introduction of GQDs.

Figure 8a shows the EQE spectra of the respective subcells for the InGaP/InGaAs/Ge triple-junction solar cell without (dashed line) and with (solid line) the deposition of GQDs. The increase of EQE efficiencies in the upper two subcells is evident after introduction of GQDs, reconfirming GQDs can enhance the collection of photocarriers and increase the photocurrent. The EQE in the bottom Ge subcell increases a little only. Figure 8b shows the EQE enhancement ratio (E/E_0_, where E and E_0_ are the EQE of subcells in the presence and absence of GQDs, respectively) as a function of the excitation wavelength. The EQE enhancement ratio of the InGaP top subcell shows a maximum wavelength at ~400 nm. The maximum wavelength agrees roughly with that of light absorption in the GQD PLE (Fig. 2d), indicating the increase of EQE correlates with the absorption of GQDs. The value of enhancement ratio of the InGaP top subcell is around 1.1 for the longer wavelength range (450–700 nm). The enhanced EQE in the wavelengths of 450–700 nm is attributed to the light trapping effect due to the increase of surface roughness^26^.

It is noted that the EQE and its enhancement ratio of the InGaAs middle subcell increase pronouncedly after incorporation of GQDs, as shown in Fig. 8a and b. This could be attributed to the luminescence coupling effect in the EQE measurement in triple-junction solar cells^27^,^28^. For triple-junction solar cells, the EQE measurement is not an easy task because each subcell cannot be contacted directly. To probe EQE of a subcell that is series-connected to other subcells, proper light bias has to be applied to other subcells^29^. However, the light biasing may produce measurement artifacts in detecting the EQE of subcells. In our case, the EQE of the InGaAs middle subcell was measured by illuminating the blue and infrared LEDs to supply light biasing to the top InGaP and bottom Ge subcell, respectively. The blue LED with photon energy greater than the bandgap energy of InGaP can be absorbed and produce the luminescence from the active layer of the top InGaP subcell. This luminescence could be reabsorbed in the middle InGaAs subcell, yielding a luminescence coupling photocurrent. Thus, the blue light, with the wavelength outside the wavelength range of the InGaAs layer, may produce an artificial phtocurrent in the InGaAs middle subcell. When GQDs are introduced on top of the top InGaP subcell, light absorption of the biased blue light would be enhanced since the maximum absorption wavelength of GQDs is in the blue spectral range (Fig. 2d). This explains the increased EQE and its enhancement ratio in the InGaAs middle subcell.

This study is just an initial effort for demonstrating carrier injection from GQDs to the III–V solar cell. Other semiconductor solar cells could improve their conversion efficiency by using GQDs as well. Recently, performances of Si solar cells have been investigated by depositing GQDs on the surface of Si solar cells^11^,^12^. After the depositon of GQDs, an enhancement of 2.94–6.1% and 2.7–14.9% has been observed in J_SC_ and η of Si solar cells, respectively. The enhanced performance in Si solar cells was explained by the energy-down-shift effect due to GQDs^12^. In our opinion, the carrier injection from GQDs should also play a role in the enhanced conversion efficiency in the GQD-deposited Si solar cells since GQDs exhibit a lower work function (~3.7 eV) than that of Si (4.2 eV)^13^. On the other hand, if GQDs are acted as the down-converter layer in our study, the maximum enhancement wavelength should occur at the wavelength of the PL peak in GQDs (~470 nm), as shown in Fig. 2(c). However, the maximum enhancement wavelength obtained form Fig. 8(b) is ~400 nm, which deviates 70 nm from the PL peak in GQDs. Therefore, the energy-down-shift is not the main effect for the enhancement of the InGaP/InGaAs/Ge triple-junction solar cell by using GQDs.

In addition to solar cells, the carrier injection technique proposed here is expected to be applied in other optoelectronic devices. According to the work function difference between GQDs and some semiconductors, GQDs could inject electrons into the semiconductors through the GQD/semiconductor interface, improving the optical and electrical properties in the active layer of devices. Performance of the optoelectronic devices such as light emitting diodes (LEDs) and photodetectors could be enhanced by the incorporation of GQDs on the device surface.

## Conclusions

A one-step method for the synthesis of GQDs by pulse laser ablation has been demonstrated successfully. The synthesized GQDs can improve the performance of InGaP/InGaAs/Ge triple-junction solar cells remarkably by incorporating them on the cell surface. The short-circuit current and conversion efficiency were increased from 11.4 to 14.9 mA/cm^2^ and 24.6 to 33.2% for incorporation of the GQDs at the concentration of 1.2 mg/ml, corresponding to a 31% and 35% enhancement compared to the cell without GQDs, respectively. On the basis of the time-resolved PL, EQE, and Kelvin probe measurements, the major contribution of efficiency enhancement in the InGaP/InGaAs/Ge triple-junction solar cell was attributed to the carrier injection from GQDs to the InGaP top subcell.

## Additional Information

**How to cite this article**: Lin, T.-N. *et al*. Enhanced Conversion Efficiency of III–V Triple-junction Solar Cells with Graphene Quantum Dots. *Sci. Rep.* **6**, 39163; doi: 10.1038/srep39163 (2016).

**Publisher's note:** Springer Nature remains neutral with regard to jurisdictional claims in published maps and institutional affiliations.
